# Atraumatic spinal needle indicates correct CSF opening pressure

**DOI:** 10.1038/s41598-022-25455-0

**Published:** 2022-12-06

**Authors:** Marcel S. Woo, Simon S. Kessner, Eckhard Schlemm, Christian Gerloff

**Affiliations:** 1 Department of Neurology, University Medical Center Hamburg-Eppendorf, Martinistr. 52, 20251 Hamburg, Germany; 2 Department of Psychosomatic Medicine and Psychotherapy, University Medical Center Hamburg-Eppendorf, Martinistr. 52, 20251 Hamburg, Germany

**Keywords:** Neurology, Diseases of the nervous system

## Abstract

The accurate assessment of cerebrospinal fluid opening pressure during spinal puncture provides important medical information in diagnosis, prognosis and therapy of several neurological conditions. However, purpose-specific spinal needle choice is debated. While atraumatic needles are associated with lower incidence of post-puncture headache and re-hospitalisation, some clinicians believe that they lack in accuracy of CSF opening pressure assessment. Our primary objective was to investigate different needle types on correctly assessing CSF opening pressure. We compared typical clinically utilised traumatic (0.9 mm outer diameter) and atraumatic (0.7 mm; 0.45 mm) spinal needles with regards to the assessment of the opening pressure in an experimental spinal puncture model testing experimental and cerebrospinal fluids in predefined pressures. Our goal was to measure the time until indicated pressure levels were correctly shown. Atraumatic needles of at least 0.7 mm diameter had a similar accuracy as traumatic needles without significant differences in time-to-equilibrium. These results were independent of protein and glucose concentration and the presence of haemoglobin. This study demonstrates that atraumatic needles can be used to accurately measure CSF opening pressure. This knowledge might guide clinicians in their choice of needle and help to reduce post-puncture headaches and re-hospitalisation.

## Introduction

### Daily routine and a common misconception

Lumbar puncture (LP) for cerebrospinal fluid (CSF) diagnostics and therapy is a daily routine for neurologists, anaesthetists, neurosurgeons, oncologists, paediatricians, and other medical clinicians worldwide. An additional procedure during LP is the assessment of the lumbar CSF opening pressure^1^, which is essential in conditions with suspected increased or decreased intracranial pressure^2^. In general, there are two types of spinal needles: traumatic and atraumatic needles, each in different gauges. It is well known that traumatic spinal needles cause more post-puncture headaches compared to atraumatic needles^3–6^.

Earlier studies that assessed flow rates and CSF opening pressure using different spinal needles showed that both atraumatic and traumatic needles can be used to accurately measure CSF pressure values^7^. However, the impact of different pathological conditions such as increased glucose or protein concentrations on assessing CSF opening pressure with traumatic or atraumatic spinal needles remain unknown.

### History of lumbar puncture

The lumbar puncture technique was first described in 1891 by the German medical doctor Heinrich Irenäus Quincke (1842–1922)^8^. His *Quincke* needle is formed similar to common medical cannulas as a metal needle with a diagonal sharp cut at its bevelled tip end. On one hand, it is very stable and stiff. On the other hand, as a drawback, its tip may mechanically cut a hole through the dura mater at the injection site during the spinal tap procedure. After discharging the needle, CSF may drain through this leak, causing post-puncture headache syndrome.

In contrast, a modernized alternative needle type was developed 1987 by Günter Sprotte, a contemporary German anaesthetist. The atraumatic *Sprotte* needle has a blunt tip designed like the top of a rocket with a cuckoo-hole-like outlet at the side^9^. Recently, similarly to the Sprotte design, Standl and colleagues introduced the Ballpen needle which comes with a pencil-like tip^10^. Another type of atraumatic needles is referred to as *Whitacre* type (which in its first formulation was designed for spinal anaesthesia, only).

The classical *Quincke* needle with its bevelled tip is commonly referred to as *traumatic* needle. In contrast, the *Sprotte* needle and derived replica are characterised by a pencil-like rotationally symmetrical tip and are commonly referred to as *atraumatic* needle.

### Post-puncture headache

A recent meta-analysis covering data from more than 31,000 patients has shown that side effects from spinal tap differ significantly between spinal needle types^3^. Lumbar punctures with traumatic needles are associated with a higher rate of post-puncture headache than with atraumatic needles (11% vs. 4.2%) and a higher rate of re-hospitalization after lumbar puncture. Therefore, careful considerations and medical indications for the use of traumatic needles are important to reduce side effects and patient suffering. This study aimed to provide conclusive and practically relevant evidence whether the needle type and size impacts the correct measurement of the CSF pressure during lumbar puncture.

To this end traumatic and atraumatic needles were compared in an experimental model with different liquids compositions and pressures to simulate physiological and pathological CSF constellations.

We hypothesized that sufficiently large atraumatic needles have a similar accuracy in assessing CSF opening pressure as traumatic needles.

## Methods

### Online survey

For an overview of the knowledge about and attitudes towards about CSF pressure measurement technique among medical doctors, we conducted a non-representative online survey (*survio.com*) and distributed the questionnaire among different hospitals in Hamburg and Northern Germany. The survey consisted of ten questions in a multiple-choice format. The questionnaire focused on the execution of lumbar puncture and the influence of needle types and sizes on CSF pressure assessment. The questions are provided in supplementary Table 1.

### Experimental setup

**Figure 1:**
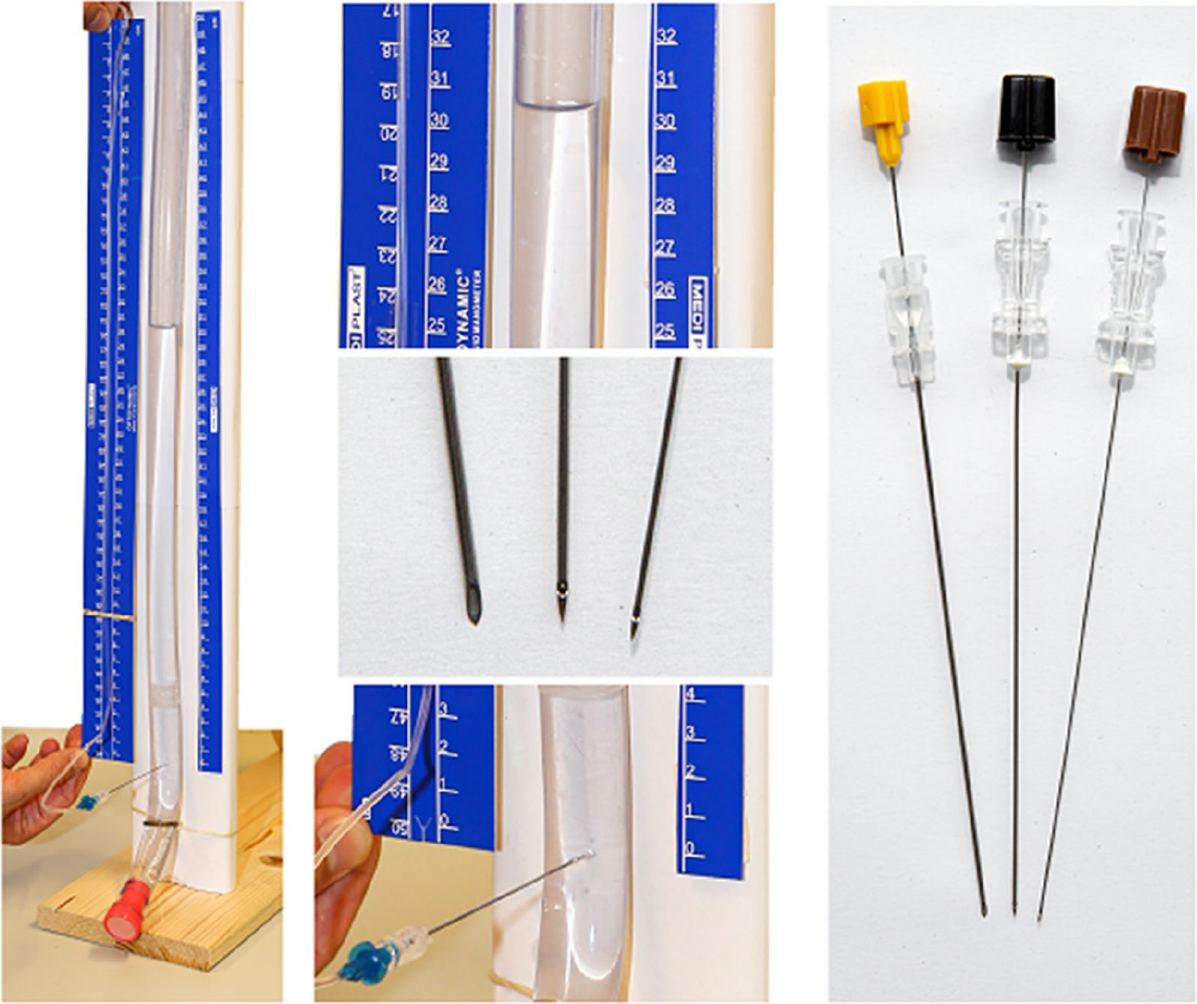
Experimental spinal puncture model and used needles. Photograph of the liquor puncture simulator (left side), the three spinal needles used in this study (right side) together with enlarged details (centre) of the peak level, the needle tips and the zero level where the needle was punctured through the tube.

For the actual experiment, an LP simulator was built, see Fig. 1. A transparent plastic hose with 16 mm outer diameter—representing the spinal dura—was vertically attached to a wooden board. After closing the lower hose opening, each different liquid was filled from the top until it reached the predefined level of water column, measured in centimetres. Three pressure levels of water columns were tested: 20 cm, 30 cm, and 50 cm.

The opening pressure of seven different liquid compositions was tested using three different spinal needles, which are used in daily clinical routine. In each test, the needle was punctured through the tube at the zero centimetre level. Subsequently, the manometer was connected to the needle, and the times to reach 80% and 100% of the predefined level of the liquid in the hose were measured. Each needle × liquid combination was tested three times. Figure 1 provides a visualization of the tested needles.

### Spinal needles

We used a traumatic needle [0.9 mm outer diameter (20 Gauge/20G); Becton Dickinson, BD Spinal Needle], and two atraumatic needles [0.7 mm (22G) and 0.45 mm (26G; Smiths medical, RapID Spinal Needle Set Pencil Point Spinal Needle)]. The reason of choice of different diameters was the clinical availability. The main goal of the study was the proof-of-principle whether or not CSF opening pressure can be assessed correctly using atraumatic needles at all. And if so, which minimal diameter should be considered. Therefore, we chose to test the spinal needles that are in use in daily routine, thus methodologically limiting the direct comparison between atraumatic and traumatic needle of the same size. Importantly, all needles were 90 mm long. We measured the pressure with a commercially available instrument (MediPlast Optidynamic Spinal Fluid Manometer).

### Artificial cerebrospinal fluid

We created artificial fluids with different viscosities to imitate clinically common conditions of human CSF by dissolving glucose (Sigma-Aldrich, cat. G8644) and milk powder (SigmaAldrich, cat. 70166) in distilled water in different concentrations. We tested five different compositions of artificial ‘CSF’: distilled water (pure H_2_O); water with normal glucose (0.7 g/L) and normal protein (0.4 g/L); water with increased glucose (7.0 g/L) and normal protein (0.4 g/L); water with normal glucose (0.7 g/L) and increased protein (10 g/L); and water with both increased glucose and protein (7.0 g/L and 10.0 g/L), respectively.

### Human cerebrospinal fluid

Additionally, we investigated two types of anonymized samples of human CSF: (a) samples of CSF with normal glucose and protein levels from patients with idiopathic intracranial hypertension and (b) CSF with high levels of haemoglobin from patients with subarachnoid haemorrhage, retrieved from external ventricular drains. For human CSF samples, only residuals that were retrieved for therapeutic purposes in routine clinical practice that would have been discarded elsewise were used. The study was registered with the local ethics committee (Ethik-Kommission der Ärztekammer Hamburg, Germany, case number #2022-300209-WF), and written informed consent was obtained from the patients. All probes were anonymized and all methods were performed in accordance with the relevant guidelines.

### Statistics

Statistical analysis and visualization were performed within the R environment^11^. We used a multiple linear regression model to test the association between needle type and measured times to reach 80% and 100% of the maximal pressure. Needle size, protein concentration, glucose concentration, and haemoglobin status were used as covariates. Subsequently, we performed ANOVA on the linear model to calculate *F* statistics.

## Results

### Online survey results

Forty-four out of 60 participants who were medical professionals of different experience levels and specialties completed the online survey. For details, see Table 1. For lumbar puncture without need for measuring the CSF opening pressure, most participants (77.3%) indicated to use atraumatic needles, whereas 20.5% indicated to use traumatic needles for this purpose and 2.2% indicated to use another technique without specification. In contrast, if CSF opening pressure was to be measured, 86.4% stated to use traumatic needles. In total, 68.1% of the participants believed that atraumatic needles could adequately assess the CSF pressure including 40.9% who stated that this would take more time. However, 31.8% of all participants still believed that the CSF pressure by lumbar punctures can be assessed correctly only by traumatic needles (Table 1).

**Table 1:** Questionnaire results.

Questionnaire with 44 participants				
**Medical specialty**				
Neurology: 91%	Neurosurgery: 3%	Paediatrics: 3%	Other: 3%	
**Level of experience**				
Head/chief: 2.3%	Senior physician: 9.3%	Specialist: 13.9%	Assistant > 4th year: 9.3%	Assistant 3rd to 4th year: 32.6%
Assistant 1st to 2nd year: 23.3%	Student: 7.0%	Other: 2.3%		
**Which needle choice for lumbar puncture WITHOUT opening pressure measurement?**				
Atraumatic: 77.3%	Traumatic: 20.5%	Other: 2.2%		
**Which needle choice for lumbar puncture WITH opening pressure measurement?**				
Atraumatic: 13.6%	Traumatic: 86.4%	Other: 0%		
**Can you use atraumatic needle for opening pressure measurement during LP?**				
Yes: 22.7%	No: 31.8%	Yes, but takes more time: 40.9%	Depends on CSF quality: 4.5%	

### Effect of needle type on time-to-equilibrium

**Figure 2:**
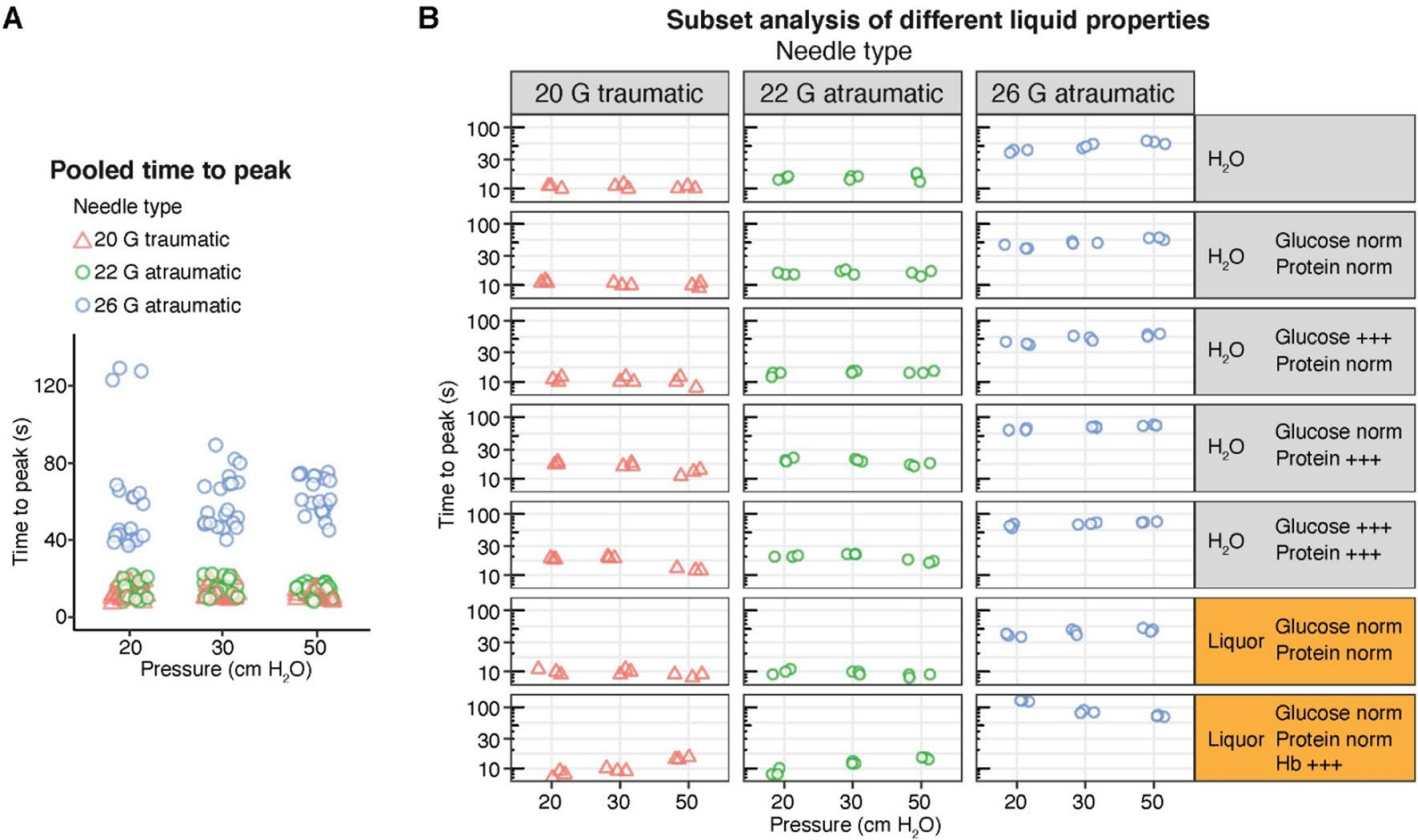
CSF opening pressure time-to-equilibrium depends from needle type and liquid. The impact of different needle types on CSF opening pressure measurements by lumbar puncture. (**A**) The time (in seconds) until the respective pressures were shown across all experimental liquid compositions. (**B**) The time (in seconds) until the respective pressures were shown, divided by needle types and different experimental liquids and CSF from healthy patients and patients who suffered from SAH.

In our experimental setup, both traumatic and atraumatic needle types reached the peak level of pressure correctly. Taken all experimental conditions together, we observed only slight but not significant (*P* = 0.07) differences in terms of the time to reach the peak level between the 20G traumatic (20 cm H_2_O, mean 12.4 s/SD 3.8 s; 30 cm H_2_O, mean 12.5 s/SD 2.8 s; 50 cm H_2_O, mean 11.2 s/SD 2.1 s) and 22G atraumatic (20 cm H_2_O, mean 14.7 s/SD 4.4 s; 30 cm H_2_O, mean 15.1 s/SD 4.1 s; 50 cm H_2_O, mean 14.8 s/SD 2.9 s). However, using the small 26G atraumatic needle it took significantly longer to reach the peak pressure (*P* < 0.0001; 20 cm H_2_O, mean 59.9 s/SD 29.8 s; 30 cm H_2_O, mean 59.8 s/SD 13.9 s; 50 cm H_2_O, mean 63.0 s/SD 9.6 s) (Fig. 2A).

### Effect of CSF composition and pressure on time-to-equilibrium

Subsequently, the effect of the different liquid compositions and pressure levels on the time-to-peak were analysed. Thus, we used water with different milk powder protein concentrations (0, 0.4 and 10 g/L), glucose levels (0, 0.7 and 7 g/L) as well as different pressure levels (20 cm H_2_O, 30 cm H_2_O, 50 cm H_2_O). We found that neither the pressure levels (*P* = 0.84) nor the glucose concentration (*P* = 0.96) significantly impacted the time to peak. In contrast, high levels of protein (*P* = 9.5e−07) prolonged the time to peak across all needles (Fig. 2B, descriptive statistics for all conditions are provided in supplementary Table 2). Notably, further analysis revealed no significant differences between 20 and 22G needles across all pressure levels and protein concentrations (20 cm H_2_O, 0 g/L *P* = 0.08, 0.4 g/L *P* = 0.07, 10 g/L *P* = 0.08; 30 cm H_2_O, 0 g/L *P* = 0.08, 0.4 g/L *P* = 0.08, 10 g/L *P* = 0.1; 50 cm H_2_O, 0 g/L *P* = 0.08, 0.4 g/L *P* = 0.1, 10 g/L *P* = 0.1).

To translate our findings into clinical practice, we compared anonymized CSF samples from patients with idiopathic intracranial hypertension (IIH) and subarachnoid haemorrhages (SAH) in our experimental setup. Similar to the results in our experimental liquid compositions we did not observe significant differences in the time to peak between the 20G traumatic (20 cm H_2_O, mean 9 s/SD 1.4 s; 30 cm H_2_O, mean 9.7 s/SD 0.8 s; 50 cm H_2_O, mean 11.5 s/SD 3.14 s) and 22G atraumatic (20 cm H_2_O, mean 9.3 s/SD 1.2 s; 30 cm H_2_O, mean 11 s/SD 1.5 s; 50 cm H_2_O, mean 11.7 s/SD 3.3 s) needles (*P* = 0.92). In contrast, the 26G (20 cm H_2_O, mean 83 s/SD 47.9 s; 30 cm H_2_O, mean 64.3 s/SD 21.6 s; 50 cm H_2_O, mean 60 s/SD 12.7 s) significantly (*P* = 3.1e−13) significantly prolonged the time to peak (Fig. 2B, descriptive statistics for all conditions are provided in supplementary Table 2). Finally, we analysed the impact of haemoglobin on time to peak. High amounts of haemoglobin significantly impaired the time to peak across all applied pressures (*P* = 0.04). Subsequent analysis revealed no significant differences between the 20G traumatic and 22G atraumatic needles across all pressure levels in human CSF with or without haemoglobin (20 cm H_2_O, Hb negative *P* = 0.99, Hb positive *P* = 0.64; 30 cm H_2_O, Hb negative *P* = 0.81, Hb positive *P* = 0.07; 50 cm H_2_O, Hb negative *P* = 0.99, Hb positive *P* = 0.62).

## Discussion

We investigated the effect of spinal needle types on the accuracy of CSF opening pressure by lumbar puncture in an experimental spine model. Our findings disprove a common belief that only traumatic needles can accurately measure correct pressure levels.

Lumbar puncture is a commonly used diagnostic and therapeutic procedure in many fields of clinical care. Post-puncture-headache is described to occur by leakage of CSF from the site of the dural tear that is created by the puncture with possible substantial burden for patients^12,13^. Due to the bevelled needle tip, lumbar punctures with traumatic needles result in a higher incidence of post-puncture headache in comparison to atraumatic needles independent of the needle size^14^. However, among healthcare professionals who routinely perform lumbar puncture, we have found that more than 30% do not think that atraumatic needles can be used for the correct assessment of opening pressure by lumbar puncture. This underlines the uncertainty of this widely used method. Beyond the needle tip shape, there might be other reasons for needle preference such as individual anatomical reasons or other factors, which exceed the scope of this survey.

The experimental setup allows to compare different needle tips and well-defined liquid compositions as well as real CSF from patients with intracranial hypertension or subarachnoid haemorrhage. Our results show that even with high protein and haemoglobin concentrations the atraumatic needle of 0.7 mm diameter (22G) assesses the same and correct pressure with negligible time delay in the range of a few seconds compared to traumatic needles with an even larger diameter. Thus, atraumatic needles of at least 0.7 mm are suitable to assess the intracranial pressure correctly and safely by lumbar puncture. However, 0.45 mm diameter needles are not suited for this purpose due to the strong time delay, especially with high protein and haemoglobin levels. Our study is limited whilst comparing different needle sizes between traumatic and atraumatic tips. Therefore, it is methodologically not possible to separate our observed effects between the needle size and the tip shape. However, these three needle types were chosen since they are routinely used for spinal taps and our main finding that 0.7 mm diameter atraumatic needle adequately show lumbar pressure without time delay is independent from comparing different needle sizes within the needle shapes. In addition, a possible technical deviation between the indicated Gauge unit of a spinal needle and the real millimetre size cannot be taken into account in this study.

This finding is highly relevant since the usage of traumatic needles is associated with an increased incidence of complications. A recent systematic meta-analysis that included data from more than 30,000 patients^3^ revealed that atraumatic needles were associated with a decreased rate of rehospitalisation for additional therapies. Importantly, they showed that the safety as well as the efficacy were comparable between traumatic and atraumatic needles.

In conclusion, our data provides evidence that atraumatic needles of at least 22G diameter correctly estimate the CSF opening pressure in comparison to traumatic 20G diameter traumatic needles with no relevant time delay across various conditions. Together with previous studies that have clearly shown the reduced incidence of post-puncture headache and other complications, these data justify the usage of atraumatic needles with at least 22G diameter for lumbar puncture if estimation of CSF opening pressure is required.

## Supplementary Information

Supplementary Tables.

## Data Availability

The data are stored by the authors and are accessible upon request with the corresponding author Dr. Dr. Simon Kessner (s.kessner@uke.de).
