# Supplementary material for: Atraumatic spinal needle indicates correct CSF opening pressure
Source: Sci Rep. 2022 Dec 6;12:21089. doi: 10.1038/s41598-022-25455-0 (PMC9726855; doi:10.1038/s41598-022-25455-0)
Supplement: Supplementary file 1 — Supplementary Tables. [file 41598_2022_25455_MOESM1_ESM.docx]

# Supplement

**Supplementary table 1. Online survey questionnaire**

| Question |  |
| --- | --- |
| 1. | Which spinal needle do you use for LP if no CSF pressure assessment is needed? |
| a) | atraumatic needle |
| b) | traumatic needle |
| c) | other |
|  |  |
| 2. | Which spinal needle do you use for LP if CSF pressure assessment is needed? |
| a) | atraumatic needle |
| b) | traumatic needle |
| c) | other |
|  |  |
| 3.. | What is your usual diameter of the spinal needle during normal LP? |
| a) | 20 gauge traumatic |
| b) | 20 gauge atraumatic |
| c) | 22 G traumatic |
| d) | 22 G atraumatic |
| e) | smaller than Gauge 22 |
| f) | not sure / do not know |
|  |  |
| 4. | What is your usually taken diameter of the spinal needle during LP with CSF pressure assessment? |
| a) | 20 gauge traumatic |
| b) | 20 gauge atraumatic |
| c) | 22 G traumatic |
| d) | 22 G atraumatic |
| e) | smaller than Gauge 22 |
| f) | not sure / do not know |
|  |  |
| 5. | How do you assess CSF pressure? |
| a) | Puncture in a sitting position, then lay the patient aside and connect the manometer. |
| b) | Puncture in a lying position and connect the manometer. |
| c) | Puncture in a sitting position and connect the manometer. |
| d) | Different procedure |
|  |  |
| 6. | Do you think that a traumatic needle and large diameter is needed for correct CSF pressure assessment? |
| a) | No, diameter does not matter. The smallest diameter displays correct pressure, too. |
| b) | No, but the smaller the diameter the longer it takes to reach peak level. |
| c) | Yes, only the traumatic large-gauge needles indicates the correct CSF pressure. |
| d) | other |
|  |  |
| 7. | What is you sex? |
| a) | male |
| b) | female |
| c) | diverse |
| d) | no comment |
|  |  |
| 8. | What is your age? |
| a) | 18 - 25 |
| b) | 26-30 |
| c) | 31-35 |
| d) | 36-40 |
| e) | >40 |
|  |  |
| 9. | What is your level of experience? |
| a) | student |
| b) | resident 1st and 2nd year |
| c) | resident 3rd to 4th year |
| d) | resident beyond 4th year |
| e) | medical specialist |
| f) | consultant |
| g) | head physician |
| h) | other |
|  |  |
| 10. | What is your specialty? |
| a) | neurology |
| b) | neurosurgery |
| c) | psychiatry |
| d) | anaesthesiology |
| e) | oncology |
| f) | paediatrics |
| g) | internal medicine |
| h) | surgery |
| i) | other |

**Supplementary table 2. Compositions of experimental fluids.**

| Pressure (cm H2O) | Protein (g/L) | Glucose (g/L) | Median | Mean | SD |
| --- | --- | --- | --- | --- | --- |
| Traumatic 20 G | | | | | |
| 20 | 0.0 | 0.0 | 11 | 10.67 | 0.58 |
| 20 | 0.4 | 0.7 | 11 | 11.33 | 0.58 |
| 20 | 0.4 | 7.0 | 11 | 11.00 | 1.00 |
| 20 | 10.0 | 0.7 | 17 | 17.67 | 1.15 |
| 20 | 10.0 | 7.0 | 18 | 18.33 | 0.58 |
| 30 | 0.0 | 0.0 | 11 | 11.00 | 1.00 |
| 30 | 0.4 | 0.7 | 10 | 10.33 | 0.58 |
| 30 | 0.4 | 7.0 | 10 | 10.67 | 1.15 |
| 30 | 10.0 | 0.7 | 16 | 16.67 | 1.15 |
| 30 | 10.0 | 7.0 | 19 | 19.33 | 0.58 |
| 50 | 0.0 | 0.0 | 10 | 10.33 | 0.58 |
| 50 | 0.4 | 0.7 | 10 | 10.00 | 1.00 |
| 50 | 0.4 | 7.0 | 10 | 10.00 | 2.00 |
| 50 | 10.0 | 0.7 | 13 | 12.67 | 1.53 |
| 50 | 10.0 | 7.0 | 12 | 12.33 | 0.58 |
| Atraumatic 22 G | | | | | |
| 20 | 0.0 | 0.0 | 15 | 15.00 | 1.00 |
| 20 | 0.4 | 0.7 | 15 | 15.33 | 0.58 |
| 20 | 0.4 | 7.0 | 14 | 13.33 | 1.15 |
| 20 | 10.0 | 0.7 | 20 | 20.33 | 1.53 |
| 20 | 10.0 | 7.0 | 20 | 20.33 | 0.58 |
| 30 | 0.0 | 0.0 | 16 | 15.33 | 1.15 |
| 30 | 0.4 | 0.7 | 17 | 16.67 | 1.53 |
| 30 | 0.4 | 7.0 | 15 | 14.67 | 0.58 |
| 30 | 10.0 | 0.7 | 20 | 20.00 | 1.00 |
| 30 | 10.0 | 7.0 | 22 | 22.00 | 0.00 |
| 50 | 0.0 | 0.0 | 17 | 16.00 | 2.65 |
| 50 | 0.4 | 0.7 | 16 | 15.67 | 1.53 |
| 50 | 0.4 | 7.0 | 14 | 14.33 | 0.58 |
| 50 | 10.0 | 0.7 | 17 | 17.00 | 1.00 |
| 50 | 10.0 | 7.0 | 17 | 17.00 | 1.00 |
| Atraumatic 26 G | | | | | |
| 20 | 0.0 | 0.0 | 43 | 41.67 | 2.31 |
| 20 | 0.4 | 0.7 | 40 | 42.00 | 3.46 |
| 20 | 0.4 | 7.0 | 42 | 42.33 | 2.52 |
| 20 | 10.0 | 0.7 | 62 | 63.33 | 2.31 |
| 20 | 10.0 | 7.0 | 64 | 64.00 | 5.00 |
| 30 | 0.0 | 0.0 | 49 | 49.67 | 4.04 |
| 30 | 0.4 | 0.7 | 49 | 49.67 | 2.08 |
| 30 | 0.4 | 7.0 | 53 | 52.00 | 4.58 |
| 30 | 10.0 | 0.7 | 69 | 69.00 | 1.00 |
| 30 | 10.0 | 7.0 | 69 | 69.67 | 3.06 |
| 50 | 0.0 | 0.0 | 58 | 57.67 | 3.51 |
| 50 | 0.4 | 0.7 | 59 | 58.00 | 2.65 |
| 50 | 0.4 | 7.0 | 59 | 58.33 | 3.06 |
| 50 | 10.0 | 0.7 | 73 | 73.33 | 1.53 |
| 50 | 10.0 | 7.0 | 74 | 74.00 | 1.00 |
